# MicroRNAs and Long Non-Coding RNAs as Regulators of NANOG Expression in the Development of Oral Squamous Cell Carcinoma

**DOI:** 10.3389/fonc.2020.579053

**Published:** 2021-02-11

**Authors:** Gašper Grubelnik, Emanuela Boštjančič, Aleksandar Aničin, Tadej Dovšak, Nina Zidar

**Affiliations:** ^1^ Institute of Pathology, Faculty of Medicine, University of Ljubljana, Ljubljana, Slovenia; ^2^ Department of Otorhinolaryngology and Cervicofacial Surgery, University Medical Centre Ljubljana, Ljubljana, Slovenia; ^3^ Faculty of Medicine, University of Ljubljana, Ljubljana, Slovenia; ^4^ Department of Maxillofacial and Oral Surgery, University Medical Centre Ljubljana, Ljubljana, Slovenia

**Keywords:** oral squamous cell carcinoma, NANOG, long non-coding RNAs, microRNAs, cancerogenesis

## Abstract

NANOG is a stem cell transcription factor that is believed to play an important role in the development of oral squamous cell carcinoma (OSCC), but there is limited data regarding the role of long non-coding RNAs (lncRNAs) and microRNAs (miRNAs) in the regulation of NANOG expression. We therefore analyzed expression of NANOG, NANOG-regulating miRNAs and lncRNAs in OSCC cancerogenesis, using oral biopsy samples from 66 patients including normal mucosa, dysplasia, and OSCC. Expression analysis of *NANOG*, *miR-34a*, *miR-145*, *RoR*, *SNHG1*, *AB209630*, and *TP53* was performed using qPCR and immunohistochemistry for NANOG protein detection. NANOG protein showed no staining in normal mucosa, very weak in low-grade dysplasia, and strong staining in high-grade dysplasia and OSCC. *NANOG*, *miR-145*, *RoR*, and *SNHG1* showed up-regulation, *TP53* and *miR-34a* showed down-regulation, and *AB209630* showed variable expression during cancerogenesis. NANOG mRNA was up-regulated early in cancerogenesis, before strong protein expression can be detected. *NANOG* was in correlation with *miR-145* and *RoR*. Our results suggest that miRNAs and lncRNAs, particularly *miR-145* and *RoR*, might be important post-transcription regulatory mechanisms of NANOG in OSCC cancerogenesis. Furthermore, NANOG protein detection has a diagnostic potential for oral high-grade dysplasia, distinguishing it from low-grade dysplasia and non-neoplastic reactive lesions.

## Introduction

Oral squamous cell carcinoma (OSCC) is associated with significant morbidity and mortality despite progress in our understanding of its etiology and pathogenesis and despite the introduction of novel treatment modalities. In 2018, OSCC accounted for more than 350,000 new diagnoses and 175,000 deaths worldwide (1–3). It is therefore necessary to discover new prognostic and predictive biomarkers. Better insight into the molecular pathogenesis of OSCC might reveal new targets for early diagnosis and the development of new therapeutic approaches (4–7).

OSCC is the result of a lengthy, highly complex multistep process, including various genetic alterations that modify signaling and also result in an altered expression of transcription factors. Morphologically, it usually develops from normal epithelium *via* hyperplasia and dysplasia to carcinoma (4–6, 8). The molecular background of cancerogenesis based on DNA and mRNA levels has been traditionally the main focus of cancer research and is now generally accepted. However, the discovery of non-coding RNAs (ncRNAs), including microRNAs (miRNAs) and long non-coding RNAs (lncRNAs), has contributed significantly to a better understanding of various physiologic and pathologic processes, including cancer. These ncRNAs regulate gene expression by various mechanisms, even though they do not encode proteins and are usually uniquely expressed in specific cell types and tissues.

The important difference between miRNAs and lncRNAs is their size, ranging between 20 and 26 nucleotides for miRNAs and 200 or more nucleotides for lncRNAs. In contrast to mRNAs, miRNAs are never capped and polyadenylated, while lncRNAs may resemble either miRNAs or mRNAs. ncRNA-mediated gene silencing executed through translational inhibition is an important biological process for cellular homeostasis in the human body. Numerous miRNAs have been shown to be significantly expressed in various cancers, including OSCC, acting as tumor suppressors and/or oncogenes. However, there is limited information about lncRNAs in OSCC and their function as sponges for expressed miRNAs (7, 9–13).

NANOG is a stem cell transcription factor, involved in the development of various human cancers including OSCC. The majority of published studies have focused on protein expression and have shown promising results, suggesting that NANOG should be considered as a diagnostic and prognostic marker of oral precancerosis and OSCC (8, 14–16). However, there is limited information regarding NANOG regulation by lncRNAs and miRNAs in OSCC development.

We therefore analyzed the expression of NANOG in oral precancerosis and OSCC in comparison to normal oral mucosa. We also analyzed ncRNAs that presumably regulate NANOG. First, we selected 13 miRNAs that could target NANOG in different cell lines using online databases TarBase v.8 (17) and miRTarBase (18), with inclusion criteria based on validation method. Next, we used literature search for selection of lncRNAs, which are experimentally validated that either sponge selected miRNAs or directly or indirectly influence *NANOG* expression (19–21). Based on the expression accuracy, we chose two miRNAs for further analysis, *i.e.*, *miR-34a* and *miR-145*, and three lncRNAs, *ROR*, *SNGH1*, and *AB209630* (7, 10, 12, 13), to reveal whether their expression supports the dynamics of the observed patterns of *NANOG* expression in OSCC cancerogenesis.

## Materials and Methods

### Patients

Our study included oral biopsy samples from 66 patients with normal mucosa, dysplasia or OSCC. Among them, 36 patients underwent surgical excision due to dysplasia, 15 patients had surgical resection due to OSCC, and in 15 patients, surgical excision was performed due to non-neoplastic lesions and served as a control group consisting of normal oral mucosa.

After surgical procedure, samples were fixed in formalin and after 24 h, embedded in paraffin (FFPE). Samples were cut at 4 µm, stained with hematoxylin and eosin (HE), and analyzed according to standard procedures.

For the purpose of our study, all biopsy samples were reviewed again, and representative samples with corresponding paraffin blocks were selected for our study. In all, slides were cut for immunohistochemical analysis of NANOG expression. After that, representative areas on HE slides were chosen and marked by ink for punching with a needle. Representative areas in patients with dysplasia comprised of LG-dysplasia and HG-dysplasia. In patients with OSCC, areas of OSCC and in the adjacent mucosa, areas of the least atypical mucosa were marked for further analysis. The areas of the least atypical mucosa showed only minor abnormalities which were consistent with LG-dysplasia. In the control group, areas of mucosa with no abnormalities were marked. Biopsy samples were divided into three groups:

Group 1 included 72 tissue samples from 36 patients with dysplasia. In each patient, two samples were included for analysis—one with LG-dysplasia and one with HG-dysplasia. 22 were males and 14 females, aged 43 to 85 years (64.47 ± 9.78). Dysplasia was located in the tongue (10 patients), floor of the mouth (13 patients), buccal mucosa (three patients), alveolar ridge (three patients), pharynx (two patients), hard palate (one patient), lip (one patient), retromolar trigonum (one patient), soft palate (one patient), and uvula (one patient).

Group 2 included 30 tissue samples from 15 patients with OSCC. In each patient, two samples were included for analysis—one with OSCC and one with LG-dysplasia (LG-dysplasia adjacent to OSCC). Nine patients had no lymph node metastases (pT1 or T2N0M0) and six patients had lymph node metastases (pT3 or T4N+M0) according to the TNM classification; 14 were males and one female, aged 37 to 74 years (60.65 ± 8.86). OSCCs were located in the tongue (eight patients), floor of the mouth (six patients) and alveolar ridge (one patient).

Group 3 included 15 biopsy samples from 15 patients with histologically normal mucosa; four were males and 11 females, aged 35 to 86 years (60.67 ± 14.89). Samples were from the buccal mucosa (six patients), lip (five patients), tongue (three patients) and retromolar trigonum (one patient).

The study was performed in accordance with the ethical guidelines for human research with no publication of identifying information or images in the manuscript and approved by the National Medical Ethics Committee, Ministry of Health, Republic of Slovenia (No 0120-106/2018/6).

### Immunohistochemical Detection of NANOG

Commercially available anti-NANOG antibody (Cell Signaling, cat no. 4903, dilution 1:200) (Merck, Kenilworth, New Jersey, USA) was used for immunohistochemistry. Automated antigen retrieval and staining (BenchMark ULTRA, Ventana, Tuscon, AZ, USA) were performed on unstained 4 µm thick slides cut from FFPE tissue blocks. Visualization of reaction was provided by peroxidase and 3,3′-diaminobenzidine incubation (OptiVIEW DAB Detection Kit, Roche, Basel, Switzerland) and after counterstaining with hematoxylin. Every run of the samples included a positive control (testicular seminoma) and negative controls omitting the primary antibody binding.

Using semi-quantitative approach, we evaluated the extent of staining (negative—score 0, below 25%—score 1, between 25 and 50%—score 2, between 50 and 75%—score 3 and above 75%—score 4). We also evaluated the intensity of staining (negative—score 0, weak—score 1, moderate—score 2, strong—score 3) and staining pattern (nuclear, cytoplasmic staining or both). We calculated the combined immunohistochemical score by multiplying the intensity and the extent scores.

### Selection of miRNAs and lncRNAs

Using online databases TarBase v.8 (17) and miRTarBase (18), we first selected 13 miRNAs that could target NANOG in different cell lines. The inclusion criteria were based on validation method. TarBase includes low- and high-throughput methods with western blot, qPCR and reporter assay being low-throughput and microarray being high-throughput. Similarly, miRTarBase distinguishes between strong evidence methods: reporter assay, western blot and qPCR and less strong validation method as are microarray, next-generation sequencing (NGS) and pSILAC. After miRNA qPCR validation described below (sections *Reverse Transcription* and *qPCR for miRNAs*), we selected miRNAs with a specific PCR product and stable expression in our samples and with low-throughput validation method in Tarbase v.8 and strong validation method in miRTarBase.

miRNA qPCR validation started by pooling samples from the same type of samples together (normal mucosa, low-grade dysplasia, high-grade dysplasia, mucosa adjacent to OSCC and OSCC) producing five pools. Next, after performing reverse transcription (RT) of pooled samples, we tested whether there is detectable expression before cycle 35, what is the efficiency of amplification and specificity. The last was obtained using melting curve that is performed after amplification using SybrGreen resulting in melting temperature specific for each amplified miRNA. It is usually lower than that for mRNAs due to short amplicons and higher than that of primer-dimers.

Analyzing samples regarding stable expression, the values should be first close among replicates and within the groups of the same sample types. For each sample, the melting curve confirmed the amplification of specific product. The sample that did not show specific PCR product was omitted from further analysis. The same approach was used for miRNAs among analyzed pools of samples.

Thus, only *miR-34a* and *miR-145* satisfy all the criteria. The results are summarized in **Table S1**.

We used literature search for selection of lncRNAs, which are experimentally validated that either sponge selected miRNAs or directly or indirectly influence *NANOG* expression. Certain lncRNAs have already been shown that have potential to influence stemness-associated transcription factors, including *NANOG* (22). Based on the literature search, we selected *RoR, SNHG1* and *AB209630*, which were experimentally validated that either sponge *miR-145* and *miR-34a* or directly or indirectly influence NANOG expression (19–21). Additionally, we analyzed the expression level of *TP53* since it is a connection point in the regulation of selected ncRNAs (23–25).

### Isolation of Total RNA

Total RNA was isolated from FFPE biopsy samples, using a 0.6-mm needle (punching) for precise tissue collection (Manual Tissue Arrayer MTA, Beecher, Estigen). Manual isolation of total RNA was performed using MagMAX FFPE DNA/RNA Ultra kit (Applied Biosystems; Thermo Fisher Scientific, Foster City, CA, USA) according to the manufacturer’s instructions with one modification. Protease digestion was performed overnight, 12 h incubation including mixing at 300 rpm for 15 s every 4 min. Reagents used were from Thermo Fisher Scientific, (Foster City, CA, USA) apart from the ethanol (Merck KGaA, Darmstadt, Germany) and the deparaffinization solution (xylene; Sigma-Aldrich; Merck KGaA, Darmstadt, Germany). The quality and concentration of isolated RNAs were assessed fluorometrically on Qubit 3.0 (Applied Biosystems; Thermo Fisher Scientific, Foster City, CA, USA). After punching, additional slide was cut and evaluated by pathologist.

### Reverse Transcription

Reverse transcription (RT) of total RNA was performed in one reaction for each sample for optimal expression comparison for mRNAs, miRNAs and lncRNAs using miScript II RT (Qiagen, Hilden, Germany) according to manufacturer’s instructions. Each 14 µl RT reaction contained 7.94 ul (12 ng/ul) of extracted total RNA, 2.8 µl HiFlex buffer, 1.4 µl 10× Nucleic mix, 1.4 µl of miScript RT enzyme and 0.46 µl of RNaze Inhibitor (Qiagen, Hilden, Germany). The reaction was incubated for 60 min at 37°C and 5 min at 95°C, using SimpliAmp Thermal Cycler (Applied Biosystems; Thermo Fisher Scientific, Foster City, CA, USA).

### Pre-Amplification and Quantitative Real Time PCR

All quantitative real time PCR (qPCR) reactions were performed using ViiA 7 Real-Time PCR System (Applied Biosystems; Thermo Fisher Scientific, Foster City, CA, USA) after RT. Each sample was done in duplicate. All qPCR reactions for efficiency testing were performed in triplicate.

#### Pre-Amplification for TaqMan Assays

Pre-amplification was performed prior to qPCR to measure the expression level of mRNAs and lncRNA, using TaqMan PreAmp mastermix (Applied Biosystems; Thermo Fisher Scientific, Foster City, CA, USA) according to the manufacturer’s instructions. Pre-amplification was performed using 6.25 µl of resulting cDNA, 12.5 µl 2× TaqMan PreAmp Mastermix (Applied Biosystems; Thermo Fisher Scientific, Foster City, CA, USA), and 6.25 µl of pooled 0.2× TaqMan Gene Expression Assays, diluted in Tris-EDTA buffer solution, pH 8.0 (Sigma-Aldrich; Merck KGaA, Darmstadt, Germany). TaqMan Gene Expression Assays used are *GAPDH* (Human GAPD; Cat. No. 4310884E), *IPO8* (Hs00183533_m1; Cat. No. 4331182), *HPRT1* (Hs99999909_m1; Cat. No. 4333768), *NANOG* (Hs04260366_g1; Cat. No. 4331182), *TP53* (Hs01034249_m1; Cat. No. 4331182) and *RoR* (Hs05054521_s1; Cat. No. 4351372) (Applied Biosystems; Thermo Fisher Scientific, Foster City, CA, USA). Cycling conditions were as follows: 10 min at 95°C and 10 cycles of 15 s at 95°C and 4 min at 60°C using SimpliAmp Thermal Cycler (Applied Biosystems; Thermo Fisher Scientific, Foster City, CA, USA).

#### qPCR for TaqMan Assays

Efficiency of qPCR reactions were calculated using pools of isolated RNAs for each group of samples. Resulting pre-amplified cDNA was diluted 5-, 25-, 125-, 625-fold for each mRNAs and 4-, 8-, 16-, 32-, 64-, 128-fold for lncRNA efficiency analysis. Reactions were performed in triplicates.

For expression analyses of mRNAs and lncRNA, TaqMan technology was used. Pre-amplified cDNA was diluted five-fold prior to qPCR. Each qPCR reaction contained 5 µl of 2× TaqMan Gene Expression Master Mix, 4.5 µl of diluted pre-amplified cDNA and 0.5 µl 20× TaqMan Gene Expression Assay (Applied Biosystems; Thermo Fisher Scientific, Foster City, CA, USA) listed in previous section. Cycling conditions were as follows: 2 min at 50°C, 10 min at 95°C and 45 cycles of 15 s at 95°C and 1 min at 60°C. *GAPDH*, *IPO8* and *HPRT1* were used as reference genes. All reactions were performed in duplicates.

#### qPCR for miRNAs

qPCR based on SYBR Green technology was performed to measure the expression level of miRNAs. Prior to qPCR, efficiency was determined using RNA pools for each group of samples. cDNA was diluted 10-, 25-, 100-, 125-, 625-, 1,000- and 3,125-fold and used in qPCR reactions as described below. Reactions were performed in triplicates.

miScript SYBR Green PCR Kit (Qiagen, Hilden, Germany) was used for miRNAs expression analysis. qPCR reaction contained 5 µl of 2× miScript SYBR Green PCR Mix, 1 µl 10× miScript Universal primer, 1 µl 10× miScript primer, 0.95 µl ddH2O, 0.05 µl of ROX dye and 2 µl of cDNA diluted 100-fold. All 10× miScript primer assays used are *SNORD61* (Hs_SNORD61_11; Cat. No. MS00033705), *SNORD95* (Hs_SNORD95_11; Cat. No. MS00033726), *miR-145* (Hs_miR-145_1; Cat. No. MS00003528), and *miR-34a* (Hs_miR-34a_1; Cat. No. MS00003318) (Qiagen, Hilden, Germany). Thermal conditions were as follows: 15 min at 95°C and 45 cycles of 15 s at 94°C, 30 s at 55°C, 30 s at 70°C. Afterwards melting curves were acquired on the SYBR channel using a ramping rate of 0.7°C/60 s for 60–95°C. *SNORD61* and *SNORD95* were used as reference genes. We tested several miRNAs that were recommended as miScript PCR Controls by the manufacturer (Qiagen). We chose *SNORD61* and *SNORD95* as normalizers since PCR products showed expression in early cycles, had specific melt curve after PCR, had acceptable efficiency of amplification, had Cq values close among replicates, within the groups and between the groups. Thus, only *SNORD61* and *SNORD95* satisfied all the criteria. All reactions were performed in duplicates.

#### qPCR for lncRNAs

SYBR Green technology was also used for qPCR to measure the expression level of lncRNAs. For efficiency determination, RNA pools of all groups of samples were reverse transcribed and resulting cDNA was diluted 4-, 8-, 16-, 32- and 64-fold. lncRNA efficiency analysis was performed in triplicate qPCR reaction as described below.

SYBR Select Master Mix (Applied Biosystems; Thermo Fisher Scientific, Foster City, CA, USA) was used for expression analysis of lncRNAs. qPCR reaction contained 5 µl of 2× SYBR Select Master Mix, 1 µl of forward and 1 µl of reverse primer and 3 µl of cDNA diluted 30-fold. All lncRNAs primers used (Sigma-Aldrich, Merck KGaA, Darmstadt, Germany) are *SNHG1* [forward: 5′-TAACCTGCTTGGCTCAAAGGG-3′; reverse: 5′-CAGCCTGGAGTGAACACAGA-3′ (26)] and *AB209630* [forward: 5′-GGGCTATTGTCCCTAAGTTGAT-3′; reverse: 5′-TGTCTTGTAGAGCATAAGGAAACC-3′ (27)]. Thermal conditions were as follows: 2 min at 50°C, 2 min at 95°C and 45 cycles of 15 s at 95°C and 1 min at 60°C. Afterwards melting curves were acquired on the SYBR channel using a ramping rate of 0.7°C/60 s for 60–95°C. *GAPDH* (Hs_GAPDH_vb.1_SG; Cat. No. QT02504278) and *PPIA* (Hs_PPIA_4_SG; Cat. No. QT01866137) were used as reference genes (Qiagen, Hilden, Germany). All reactions were performed in duplicates.

### Validation of the Expression of NANOG, TP53, Selected miRNAs and lncRNAs in the Oral Squamous Cell Carcinoma From The Cancer Genome Atlas RNA Sequencing Datasets

To validate the expression of analyzed transcripts we used the RNA sequencing data of 523 head and neck squamous cancer (HNSCC) samples and 44 normal mucosa adjacent to HNSCC from The Cancer Genome Atlas. Of these, 329 correspond to OSCC and 32 to normal mucosa adjacent to OSCC. No sample of precancerous lesions was found in database. The data were obtained from cBioPortal interactive online database. In all, mRNA, lncRNA and miRNA experiments, level 3 data was used, which contains normalized gene counts and read per million mapped miRNA isoforms, respectively. The data were represented as reads per million in box plots.

### Statistical Analysis

For mRNA, miRNA and lncRNA expression analysis, data were analyzed according to Latham (28). For each sample, geometric mean of Cqs of reference genes for mRNAs, miRNAs or for lncRNAs were subtracted from mRNAs, miRNAs or lncRNAs, to obtain ΔCq for statistical analysis.

Comparison of relative quantification of mRNAs, miRNAs and lncRNAs (ΔCq) between independent group of samples was performed using Mann–Whitney U test. Correlation between mRNAs, lncRNAs and miRNAs was performed using Spearman Rank correlation coefficient test. Statistical analysis of data was performed using IBM SPSS Statistics 24 software (SPSS Inc., Chicago, IL, USA). Differences were considered as significant using cut-off p <0.05 (two-tailed). ΔΔCq was used for calculating fold change using log2-ΔΔCq for graphical presentation.

## Results

### Immunohistochemical Expression of NANOG Protein

Thirty-six cases of high-grade dysplasia (HG-dysplasia), 31 cases of low-grade dysplasia (LG-dysplasia), 15 cases of OSCC, 15 cases of LG-dysplasia adjacent to OSCC, and 15 cases of normal oral mucosa were analyzed for NANOG protein expression.

**Figure 1:**
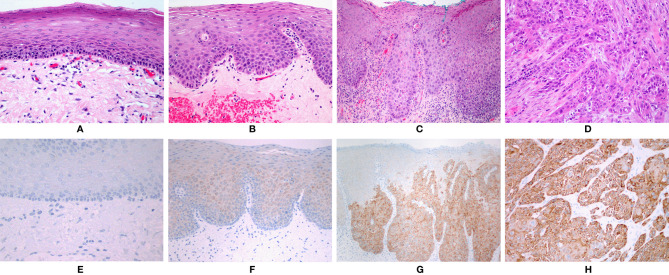
**(A)** Normal oral mucosa. **(B)** Low-grade dysplasia. **(C)** High-grade dysplasia. **(D)** Squamous cell carcinoma. **(A–D)**: HE, orig. magnification, 100×. **(E)** No immunohistochemical expression of NANOG in normal mucosa. **(F)** Faint staining in low-grade dysplasia. **(G)** Strong staining in areas with high-grade dysplasia. **(H)** Strong staining in squamous cell carcinoma. **(E–H)**: Immunohistochemistry, orig. magnification, 100×.

No staining was found in normal mucosa samples. Positive reaction was found in 34 (94.44%) cases of HG-dysplasia and in all 15 (100%) cases of OSCC. HG-dysplasia cases showed moderate staining in 11 (30.56%) and strong staining in 23 (63.89%) cases. Six (40%) OSCC cases showed moderate staining and nine (60%) cases showed strong staining. Very weak staining was found in LG-dysplasia in 29 (93.55%) cases and in 13 (86.67%) cases of LG-dysplasia adjacent to OSCC. In dysplasia, positive reaction was present in all cells. In OSCC, the extent of positive reaction was 50–75% of cells in 2 OSCC samples (13.33%) and above 75% of cells in 13 OSCC samples (86.67%). The combined immunohistochemical scores are presented in **Table 1**. Representative examples of immunohistochemical staining are shown in **Figure 1**.

**Table 1:** Combined immunohistochemical score for NANOG immunostaining.

	Combined immunohistochemical score (mean ± SD)
Normal mucosa	0
Low-grade dysplasia	3.73 ± 1.00
High-grade dysplasia	10.4 ± 3.2
Squamous cell carcinoma	9.87 ± 2.87
Low-grade dysplasia adjacent to carcinoma	3.47 ± 1.36

NANOG was detected in the cytoplasm of epithelial cells only; no nuclear staining was observed (**Figure 1**). Positive control (testicular seminoma) showed only nuclear staining in all tumor cells.

### Expression of mRNAs, miRNAs, and lncRNAs

#### Expression of mRNAs, miRNAs, and lncRNAs in Low-Grade Dysplasia, Hight GadeDysplasia, and Oral Squamous Cell Carcinoma in Comparison to Normal Mucosa

*NANOG*, *TP53*, *miR-34a*, and *miR-145* expression was detected in all of the tested samples in normal mucosa (n = 15), LG-dysplasia (n = 31), HG-dysplasia (n = 36), and in OSCC (n = 15). However, expression of lncRNAs was not detected in all samples; *RoR* was detected in three samples of normal mucosa, 12 LG-dysplasia, 22 HG-dysplasia samples, and in five OSCC samples. *SNHG1* was detected in 13 normal mucosa samples, 20 LG-dysplasia, 20 HG-dysplasia, and in six OSCC samples. *AB209630* was detected in 15 normal mucosa samples, 26 LG-dysplasia, 31 HG-dysplasia, and in six OSCC samples.

Comparison of normal mucosa with LG-dysplasia revealed up-regulation of *NANOG* (1.74-fold; p < 0.001), *miR-145* (1.49-fold; p = 0.038), *RoR* (143.93-fold; p = 0.009), and *SNHG1* (1.53-fold; not significant) and similar expression (slight down-regulation) of *TP53* (1.12-fold; not significant), *miR-34a* (1.15-fold; not significant), and *AB209630* (1.17-fold; not significant) in LG-dysplasia.

Comparison of normal mucosa with HG-dysplasia revealed up-regulation of *NANOG* (1.65-fold; p = 0.002), *miR-145* (1.34-fold; not significant), *RoR* (219.70-fold; p = 0.006) and *SNHG1* (2.42-fold; p = 0.005) and down-regulation of *TP53* (1.31-fold; not significant), *miR-34a* (1.24-fold; not significant), and *AB209630* (1.44-fold; p = 0.043) in HG-dysplasia.

Comparison of normal mucosa with OSCC revealed up-regulation of *NANOG* (1.29-fold; not significant), *miR-145* (2.33-fold; p = 0.007), *RoR* (231.56-fold; p = 0.025), *AB209630* (27.93-fold; p < 0.001), and *SNHG1* (3.26-fold; p = 0.007) and down-regulation of *TP53* (1.98-fold; p = 0.001) and *miR-34a* (1.98-fold; p < 0.001) in OSCC.

**Figure 2:**
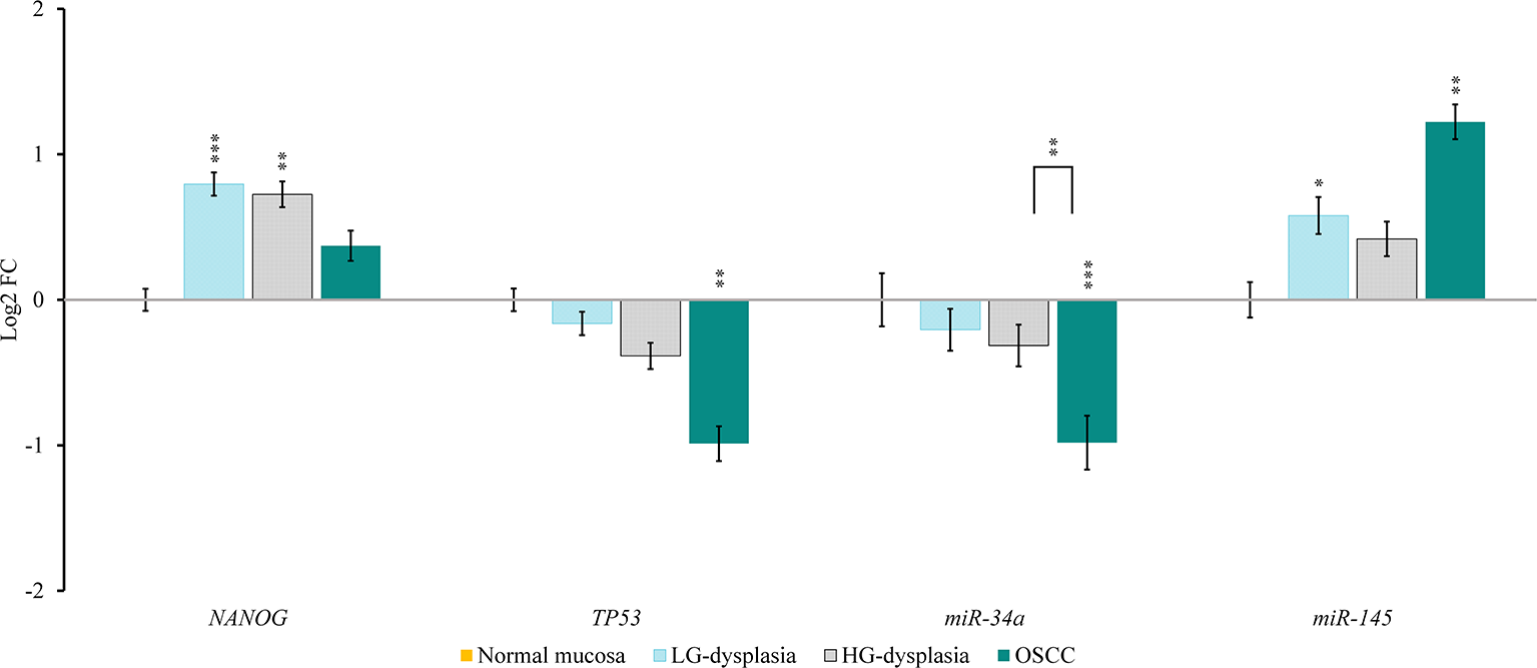
Relative expression levels of tested mRNAs and miRNAs in low-grade dysplasia (LG-dysplasia) (n = 31), high-grade dysplasia (HG-dysplasia) (n = 36) and in squamous cell carcinoma (OSCC) (n = 15) as compared with normal mucosa (n = 15) are shown as log2 of fold change. The bars represent the means ± standard deviation. Statistical comparison of relative expression levels in HG-dysplasia compared to OSCC is also shown as a line above the bars of HG-dysplasia and OSCC. Data were assessed with the Mann–Whitney U test, where p <0.05 was considered to be statistically significant (*p < 0.05, **p < 0.01, ***p < 0.001).

**Figure 3:**
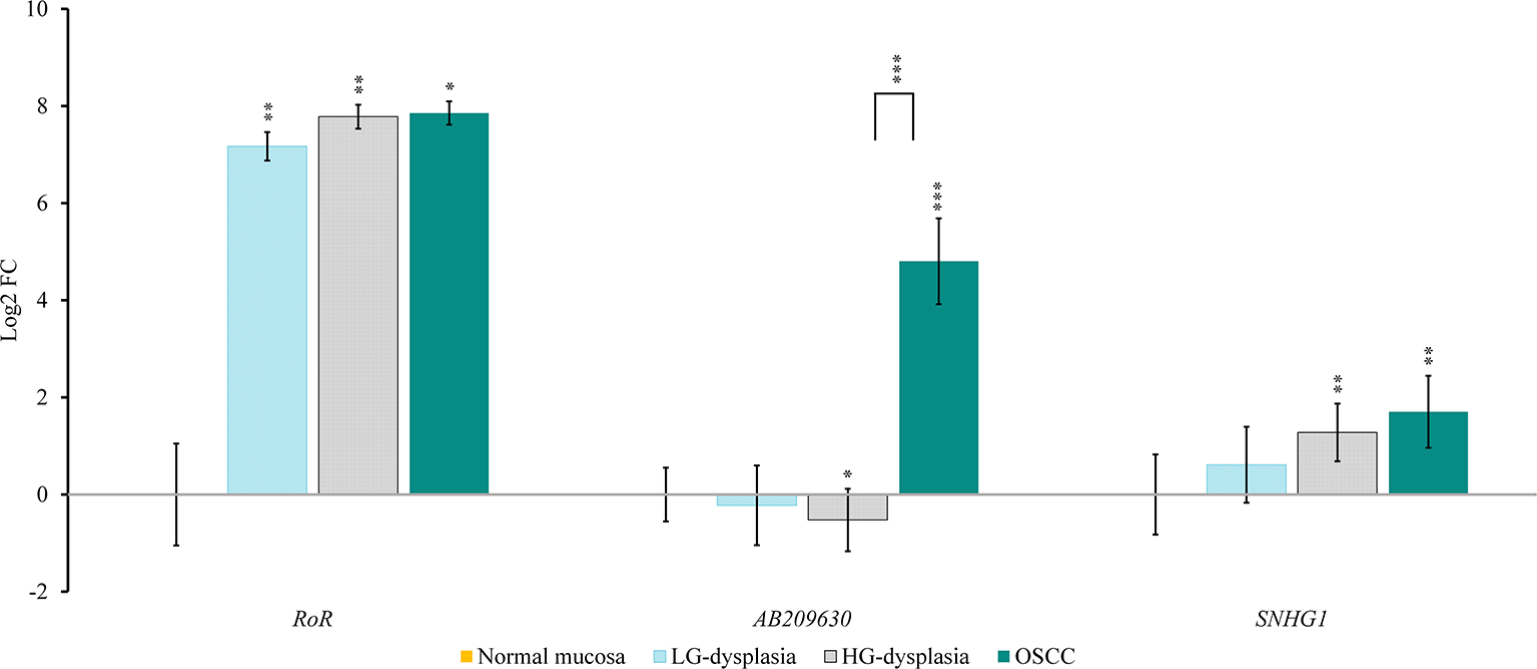
Relative expression levels of tested lncRNAs in low-grade dysplasia (LG-dysplasia) (n = 31), high-grade dysplasia (HG-dysplasia) (n = 36) and in squamous cell carcinoma (OSCC) (n = 15) as compared with normal mucosa (n = 15) are shown as log2 of fold change. The bars represent the means ± standard deviation. Statistical comparison of relative expression levels in HG-dysplasia compared to OSCC is also shown as a line above the bars of HG-dysplasia and OSCC. Data were assessed with the Mann–Whitney U test, where p <0.05 was considered to be statistically significant (*p < 0.05, **p < 0.01, ***p < 0.001).

Results are presented as log2 of fold change in **Figure 2** (for mRNAs and miRNAs) and in **Figure 3** (for lncRNAs).

**Figure 4:**
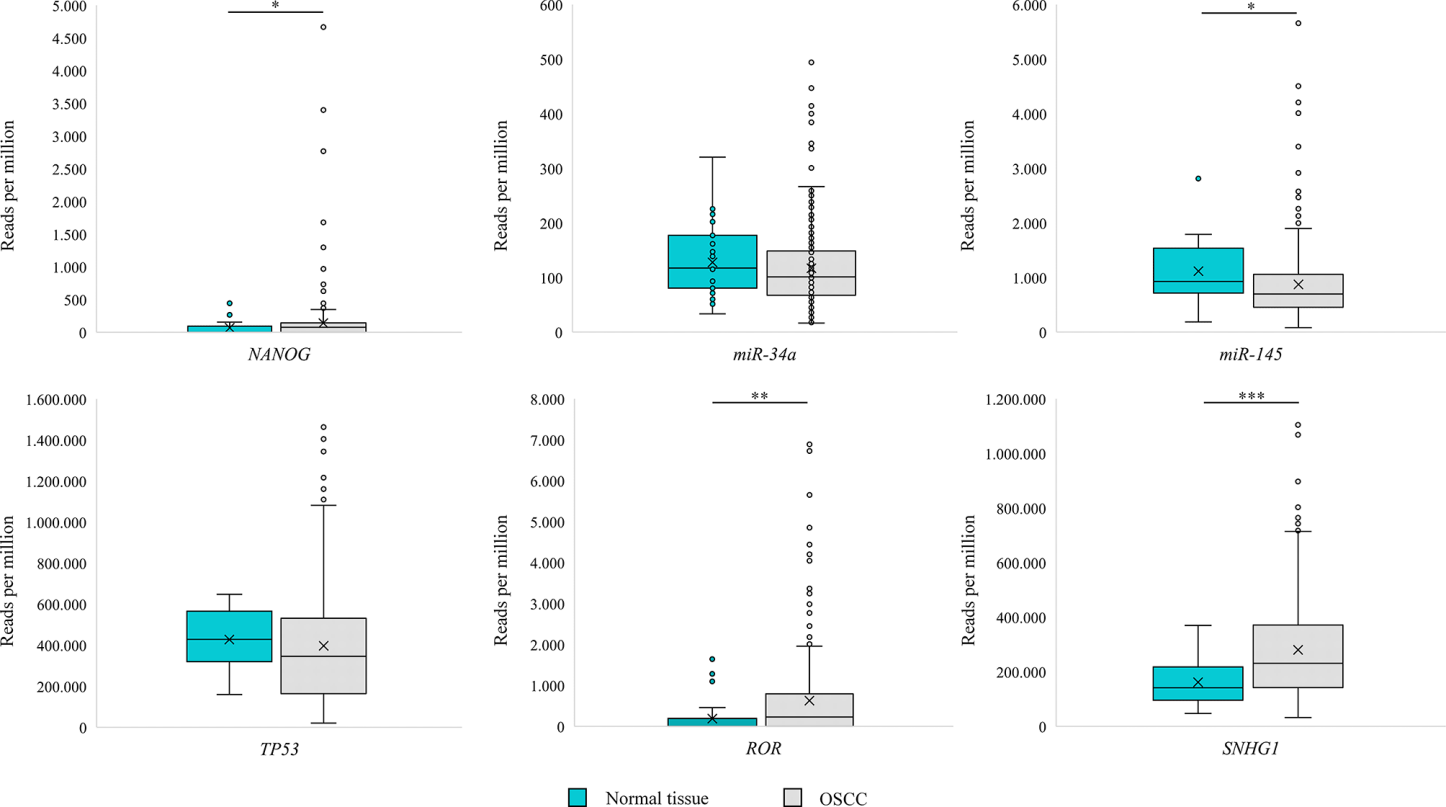
Box Plot representing expression of NANOG, TP53, miR-34a, miR-145, RoR and SNGH1 in oral squamous cell carcinoma (OSCC) and morphologically normal mucosa adjacent to OSCC in samples from The Cancer Genome Atlas. Data were assessed with the Mann–Whitney U test, where p <0.05 was considered to be statistically significant (* p < 0.05, ** p < 0.01, *** p < 0.001).

Analysis of OSCC (n = 329) from TCGA dataset revealed transcripts for *miR-34a*, *miR-145*, *TP53*, *SNHG1* in all samples, whereas *NANOG* and *RoR* were detected in 198 (60%) and 200 (61%) samples, respectively. Results are presented as reads per million in **Figure 4**.

#### Expression of mRNAs, miRNAs, and lncRNAs in Normal Mucosa in Comparison to Low Grade Dysplasia Adjacent to Oral Squamous Cell Carcinoma

*NANOG*, *TP53*, *miR-34a*, and *miR-145* expression was detected in all tested samples in LG-dysplasia adjacent to OSCC (n = 15). However, expression of lncRNAs was not detected in all samples; *RoR* was detected in five, *SNHG1* in four, and *AB209630* in eight samples of LG-dysplasia adjacent to OSCC.

Expression in LG-dysplasia adjacent to OSCC revealed up-regulation of *NANOG* (1.32-fold; not significant), *miR-145* (1.85-fold; p = 0.004), *RoR* (59.99-fold; p = 0.025), *SNHG1* (4.91-fold; p = 0.024), and *AB209630* (24.93-fold; p < 0.001), as well down-regulation of *TP53* (2.11-fold; p = 0.002) and *miR-34a* (1.77-fold; p < 0.001) in comparison to normal mucosa (data not shown).

Analysis of normal tissue adjacent to OSCC (n = 32) from TCGA dataset revealed transcripts for *miR-34a*, *miR-145*, *TP53*, *SNHG1* in all samples, whereas *NANOG* and *RoR* were detected in 13 (40%) and 11 (34%) samples, respectively. Results are presented as reads per million in **Figure 4**. Using Mann–Whitney U test we observed a significant difference in reads per million for *miR-145* (p = 0.047), *SNHG1* (p < 0.001)*, NANOG* (p = 0.043), and *RoR (*p = 0.001) between OSCC (n = 329) and normal tissue adjacent to OSCC.

#### Relative Quantification of mRNAs, miRNAs, and lncRNAs of Hight Grade Dysplasia Compared to Oral Squamous Cell Carcinoma

In OSCC in comparison to HG-dysplasia we showed up-regulation of *miR-145* (1.75-fold; p = 0.060), *AB209630* (40.18-fold; p < 0.001), and *SNHG1* (1.45-fold; not significant). *NANOG* (1.28-fold; not significant), *TP53* (1.45-fold; p = 0.053) and *miR-34a* (1.59-fold; p = 0.005) were down-regulated in OSCC. *RoR* showed similar expression (1.05-fold; not significant). Results are presented as log2 of fold change in **Figure 2** (for mRNAs and miRNAs) and in **Figure 3** (for lncRNAS).

#### Expression of mRNAs, miRNAs, and lncRNAs in Low Grade Dysplasia in Comparison to Low Grade Dysplasia Adjacent to Oral Squamous Cell Carcinoma

LG-dysplasia adjacent to OSCC revealed up-regulation of *miR-145* (1.24-fold; not significant), *SNHG1* (3.21-fold; p = 0.063) and *AB209630* (29.08-fold; p < 0.001) and down-regulation for *NANOG* (1.31-fold; not significant), *TP53* (1.89-fold; p = 0.002), *miR-34a* (1.54-fold; p = 0.001), and *RoR* (2.40-fold; not significant) in comparison to LG-dysplasia (data not shown).

#### Correlation between expression of mRNAs, miRNAs and lncRNAs

Spearman rank-order correlation (n = 112) revealed strong positive correlation between *NANOG* and *miR-145* (r_s_ = 0.315, p = 0.001) and *RoR* (r_s_ = 0.684, p < 0.001). We also observed strong positive correlation between the expression of *RoR* and *miR-145* (r_s_ = 0.480, p = 0.001) and strong negative correlation between the expression of *TP53* and *AB209630* (r_s_ = −0.392, p < 0.001) and *SNHG1* (r_s_ = −0.272, p = 0.031). There was no correlation between other tested mRNAs, miRNAs, and lncRNAs.

## Discussion

Our results suggest that NANOG plays an important role in the process of OSCC cancerogenesis. Immunohistochemical analysis showed no expression of NANOG protein in normal oral mucosa, very weak staining in LG-dysplasia, and strong staining in HG-dysplasia and OSCC. The intensity of staining correlated strongly with the severity of atypia. Strong expression of the NANOG protein in both HG-dysplasia and OSCC supports the true neoplastic nature of HG-dysplasia. The marked difference in intensity between LG-dysplasia and HG-dysplasia indicates a potential use of NANOG immunohistochemistry in diagnostic work, enabling a distinction between reactive lesions and LG-dysplasia on the one hand, and HG-dysplasia as a true precancerous lesion on the other.

Similarly, previous studies have also shown negligible expression of NANOG protein in normal mucosa of the head and neck, and up-regulation in the early stages of OSCC cancerogenesis (8, 14). Moreover, NANOG expression was significantly higher in OSCC and adjacent mucosa than in normal mucosa (29). NANOG protein was detected in 60% of laryngeal dysplasias, with 27% of dysplasias showing strong NANOG immunostaining. Five years after the initial diagnosis, only 20% of patients with negative to moderate NANOG expression and 55% of patients with strong NANOG expression developed laryngeal cancer (14). Similar results have been described for oral mucosa. In oral dysplasia, NANOG protein expression was significantly correlated with higher risk of progression to invasive carcinoma and higher cancer incidence with a stronger cytoplasmic reaction (8). Other studies also showed NANOG protein expression to increase with the grade of dysplasia, and NANOG protein expression in 31–100% of OSCC samples with immunostaining of various intensity (14, 15, 29–35).

Our study also showed that, in contrast to protein expression, mRNA was detected in all samples, from normal oral mucosa to dysplasia and carcinoma, showing the lowest values in normal mucosa. The only significant difference in mRNA expression was observed between normal mucosa and dysplasia, suggesting that *NANOG* mRNA expression is up-regulated early in the process of oral cancerogenesis, before strong protein expression can be detected.

There are limited data regarding *NANOG* mRNA expression in oral precancerosis and OSCC. A previous study reported presence of *NANOG* mRNA in 100% of OSCC samples and in 91.7% of non-tumoral margins. However, expression was significantly higher in non-tumoral margins compared to OSCC (33). Similar results of *NANOG* mRNA expression in RNA later-stored OSCC samples (n = 90) showed expression in 100% of OSCC samples and in 88.9% of corresponding normal tissue samples (36). *NANOG* mRNA expression was significantly higher in poorly and moderately differentiated OSCC than in well-differentiated OSCC (37). *In silico* analysis of the *NANOG* mRNA expression in a cohort of head and neck SCC (n = 530) using data from The Cancer Genome Atlas (TCGA) showed a significantly increased mRNA level in primary tumors compared to normal tissue. However, in an OSCC cohort (n = 172), only 2.9% of samples revealed *NANOG* mRNA up-regulation compared to normal tissue (8), similarly to our results of TCGA analysis.

The finding of mRNA in all samples and strong protein expression only in HG-dysplasia and carcinoma strongly suggest that *NANOG* expression is regulated at the post-transcriptional level. We therefore analyzed the expression of ncRNAs that presumably regulate NANOG. We found *miR-34a* to be down-regulated; its expression progressively decreased similarly to *NANOG* expression. Expression levels were the highest in normal mucosa, and progressively decreased from LG-dysplasia to HG-dysplasia, and were the lowest in OSCC. Expression of *miR-34a* was significantly down-regulated in OSCC compared to HG-dysplasia. These results indicate that *miR-34a* is down-regulated during OSCC carcinogenesis and might thus enable *NANOG* up-regulation. Our results are in accordance with previous studies that have shown up-regulation of *NANOG* and down-regulation of *miR-34a* in OSCC compared to controls (38). *miR-34a* might also regulate *NANOG* through TP53 protein, which had the same expression pattern in our samples as *miR-34a* and can directly induce the expression of pro-apoptotic *miR-34a* (23, 24, 38, 39).

*miR-145* expression analysis showed a different pattern; it was low in normal mucosa, and slightly up-regulated in LG-dysplasia and HG-dysplasia. A significant up-regulation of *miR-145* was found in OSCC. *miR-145* was up-regulated in OSCC compared to HG-dysplasia. However, these results are not in accordance with previous studies, which have shown lower expression levels of *miR-145* in OSCC and OSCC cell lines compared to normal mucosa (40–42). Furthermore, we observed a strong positive correlation between the expression of *miR-145* and *NANOG*. Consistent with our observed reverse pattern of *NANOG* expression compared to *miR-145*, a study on human embryonic stem cells and cervical cells showed that *miR-145* could down-regulate *NANOG* (19, 43). These results suggest that *miR-145* might play an important role in oral cancerogenesis.

We also analyzed lncRNAs that might regulate the investigated miRNAs. *RoR* expression levels were the lowest in normal mucosa and significantly up-regulated in LG-dysplasia, and even more in HG-dysplasia and OSCC. There was no difference in expression of *RoR* between OSCC and HG-dysplasia. We also observed a strong positive correlation between *RoR*, *miR-145* and *NANOG*. *RoR* is a long intergenic ncRNA. It is a regulator of reprogramming, functioning as a competing endogenous RNA, thus playing an important role in cancerogenesis. Previous studies have demonstrated a significant up-regulation of *RoR* and down-regulation of *miR-145* in undifferentiated oral tumors (44). It has been suggested that *RoR* could sponge *miR-145*, leading to *NANOG* up-regulation. The correlation between *miR-145* and *NANOG* supports this suggestion. *RoR* transcription can be trans-activated with *TP53* and, in turn, it can suppress *TP53* during DNA damage by suppressing p53 translation, thus inhibiting *TP53*-mediated cell cycle arrest and apoptosis in OSCC (25, 44, 45). In human embryonic cell lines, it has been demonstrated that *RoR* can protect *NANOG* from miRNA-mediated degradation and regulate *miR-145* and NANOG indirectly by post-transcriptional fine-tuning (19). Our results showed a similar *RoR* up-regulation in HG-dysplasia and in OSCC, and these patterns were similar to *miR-145* up-regulation and inverse to *NANOG* progressively decreasing expression in OSCC cancerogenesis.

The next lncRNA we analyzed was *AB209630*. Its expression levels were the lowest in HG-dysplasia and LG-dysplasia compared to normal mucosa, whereas in OSCC, they were significantly increased. *AB209630* was up-regulated in OSCC compared to HG-dysplasia. There are very limited data on *AB209630* expression in OSCC, and it has only recently been described in hypopharyngeal SCC. It was reported to be down-regulated in tumor tissue compared to adjacent tissue, and these data suggest that increased expression might inhibit or stimulate hypopharyngeal SCC development (27). Similarly, studies on hepatocellular carcinoma and on pancreatic ductal adenocarcinoma showed *AB209630* down-regulation in tumor samples compared to adjacent tissue (46, 47). Published data indicate a possible correlation between the expression of *NANOG* and *AB209630* in human pancreatic cancer cells (21). To the best of our knowledge, this is the first study on the expression of *AB209630* in OSCC carcinogenesis.

The last lncRNA we analyzed was *SNHG1*. Its expression levels were the lowest in normal mucosa and up-regulated in LG-dysplasia, with increased expression in HG-dysplasia and again with a small decrease in OSCC, suggesting up-regulation of *SNHG1* during OSCC carcinogenesis. Expression of *SNHG1* was also up-regulated when OSCC was compared to HG-dysplasia. *SNHG1* is a small nucleolar RNA host gene 1, a novel lncRNA that is increased in various human cancers, but there is no data regarding OSCC. The regulatory mechanisms of *SNHG1* are not yet known. *SNHG1* is believed to function as a sponge for *miR-145*, causing its down-regulation (20). *SNHG1* has been reported to be up-regulated in non-small cell lung cancer; in colorectal carcinoma and laryngeal carcinoma, it has been associated with cancer stage, the presence of metastasis, and a worse prognosis (20, 48–52).

**Figure 5:**
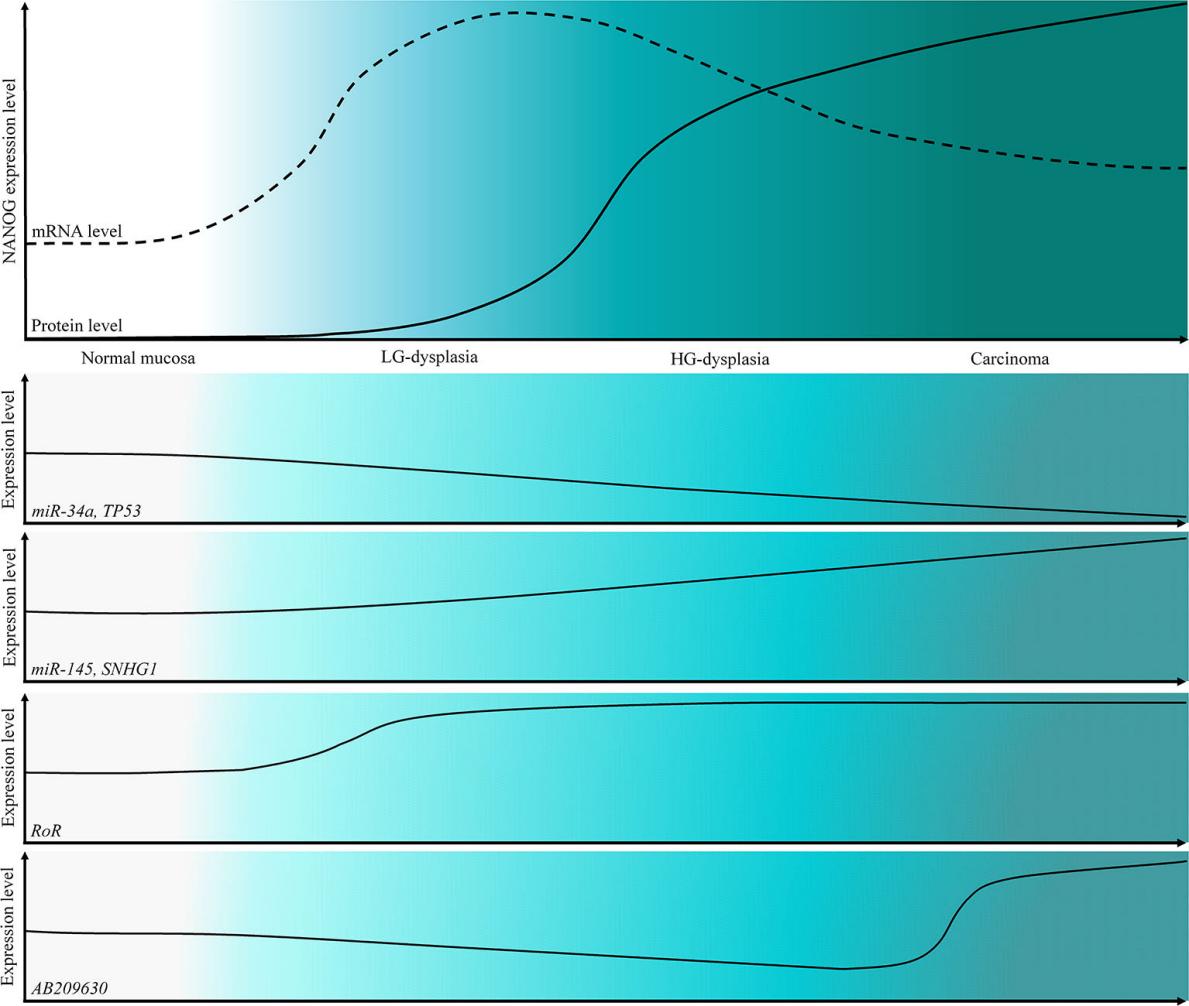
Schematic representation of the expression levels of NANOG protein and mRNA and a schematic representation of the expression levels of *TP53* and tested ncRNAs in OSCC cancerogenesis progression.

The main weakness of our study is the lack of functional validation. However, our results indicate that miRNAs and lncRNAs, particularly *miR-145* and *RoR*, might be among important regulatory mechanisms of NANOG in dysplasia and cancer development of oral cavity, indicating their potential role as biomarkers and therapeutic targets. Summary of our results are presented in **Figure 5** on a schematic representation of the expression levels of NANOG, *TP53* and ncRNAs.

In conclusion, our results further support the postulated role of NANOG in oral cancerogenesis. Being an embryonic stem cell transcription factor, protein expression is silenced before birth, and it is strongly re-expressed in HG-dysplasia and OSCC. The expression of mRNA in all samples, even in normal mucosa, and strong expression of the protein only in HG-dysplasia and OSCC point to a crucial role of post-transcriptional regulatory mechanisms governing NANOG expression in adults. Our results also strongly suggest that adjacent mucosa to OSCC should not be used for molecular analysis as “normal tissue”, consistent with the concept of field cancerization (53) even though it histologically seems normal. According to our results, only true independent samples of normal mucosa are appropriate for gene expression analysis.

## Data Availability Statement

The datasets used and analyzed during the current study are available from the corresponding author on reasonable request.

## Ethics Statement

The study involving human participants was reviewed and approved by the National Medical Ethics Committee, Ministry of Health, Republic of Slovenia (No 0120-106/2018/6). The patients/participants provided their written informed consent to participate in this study.

## Author Contributions

NZ, GG, and EB conceptualized the study. GG, AA, TD, EB, and NZ developed the methodology. EB and GG were in charge of the software. GG and EB validated the study. GG, AA, TD, EB, and NZ conducted the formal analysis. GG and EB conducted the investigation. NZ provided the resources. GG, EB, and NZ conducted the data curation. GG and EB wrote and prepared the original draft. GG, EB, AA, TD, and NZ wrote, reviewed, and edited the manuscript. GG and EB conducted the visualization. EB and NZ supervised the study. All authors contributed to the article and approved the submitted version.

## Funding

This research was funded by the Slovenian Research Agency (ARRS) under PhD thesis grant for young researcher GG and under research core funding No. P3-0054.

## Conflict of Interest

The authors declare that the research was conducted in the absence of any commercial or financial relationships that could be construed as a potential conflict of interest.
