# Supplementary material for: MicroRNAs and Long Non-Coding RNAs as Regulators of NANOG Expression in the Development of Oral Squamous Cell Carcinoma
Source: Front Oncol. 2021 Feb 11;10:579053. doi: 10.3389/fonc.2020.579053 (PMC7906007; doi:10.3389/fonc.2020.579053)
Supplement: Supplementary file 1 [file Table_1.docx]

**Table S1.** Selection of miRNAs targeting *NANOG* mRNA.

| ***miRNAs*** | **TarBase v.8** | **miRTarBase** | **qPCR validation** |
| --- | --- | --- | --- |
| *miR-27a* | High-throughput: Microarray | / | Accurate |
| ***miR-34a*** | **Low-throughput: Western blot** | **Strong: western, reporter assay** | **Accurate** |
| *miR-34b* | Low-throughput: Western blot | Strong: western, reporter assay | Unspecific |
| *miR-34b* | Low-throughput: Western blot | Strong: western, reporter assay | Unspecific |
| *miR-99b* | Low-throughput: Reporter assay | / | Unspecific |
| *miR-128* | / | Strong: western, reporter assay, qPCR | Accurate |
| *miR-134* | Low-throughput: not determined | Strong: western, qPCR | / |
| *miR-137* | Low-throughput: qPCR | / | Unspecific |
| ***miR-145*** | **Low-throughput: Reporter assay** | **Strong: western** | **Accurate** |
| *miR-181a* | / | Strong: western | Unspecific |
| *miR-302* | / | Strong: western, reporter assay, qPCR | Unspecific |
| *miR-320* | / | Less strong: NGS | / |
| *miR-335* | High-throughput: Microarray | Less strong: microarray | Accurate |
